# Angiopoietin-1 variant reduces LPS-induced microvascular dysfunction in a murine model of sepsis

**DOI:** 10.1186/cc11666

**Published:** 2012-10-04

**Authors:** Alessio Alfieri, Jay J Watson, Richard A Kammerer, Mohammed Tasab, Pavlos Progias, Kimberly Reeves, Nicola J Brown, Zoe L Brookes

**Affiliations:** 1 Microcirculation Research Group, Faculty of Medicine, Dentistry and Health, University of Sheffield, Sheffield S10 2RX, UK; 2 Laboratory of Biomolecular Research, Paul Scherrer Institut, CH-5232 Villigen PSI, Switzerland; 3 Wellcome Trust Centre for Cell-Matrix Research, Faculty of Life Sciences, University of Manchester, Manchester M13 9PT, UK

## Abstract

**Introduction:**

Severe sepsis is characterised by intravascular or extravascular infection with microbial agents, systemic inflammation and microcirculatory dysfunction, leading to tissue damage, organ failure and death. The growth factor angiopoietin (Ang-1) has therapeutic potential but recombinant Ang-1 tends to aggregate and has a short half-life *in vivo*. This study aimed to investigate the acute effects of the more stable Ang-1 variant matrilin-1-angiopoietin-1 (MAT.Ang-1) on the function of the microcirculation in an experimental model of sepsis, and whether any protection by MAT-Ang-1 was associated with modulation of inflammatory cytokines, angiogenic factors or the endothelial nitric oxide synthase (eNOS)-Akt and vascular endothelial (VE)-cadherin pathways.

**Methods:**

Aluminium window chambers were implanted into the dorsal skinfold of male C3H/HeN mice (7 to 10 weeks old) to expose the striated muscle microcirculation. Endotoxemia was induced by intraperitoneal injection of lipopolysaccharide (LPS, 1 mg/kg at 0 and 19 hours). MAT.Ang-1 was administered intravenously 20 hours after the onset of sepsis. Microcirculatory function was evaluated by intravital microscopy and Doppler fluximetry.

**Results:**

Endotoxemia resulted in macromolecular leak, which was ameliorated by MAT.Ang-1 post-treatment. LPS induced a dramatic reduction in tissue perfusion, which was improved by MAT.Ang-1. Proteome profiler array analysis of skeletal muscle also demonstrated increased inflammatory and reduced angiogenic factors during endotoxemia. MAT.Ang-1 post-treatment reduced the level of IL-1β but did not significantly induce the expression of angiogenic factors. MAT.Ang-1 alone did not induce leak or increase angiogenic factors but did reduce vascular endothelial growth factor expression in controls.

**Conclusion:**

Administration of MAT.Ang-1 after the onset of sepsis protects the microcirculation from endotoxemia-induced vascular dysfunction through reducing inflammation but without pro-angiogenic actions, thus representing a novel, potential pharmacotherapeutic agent for the treatment of sepsis.

## Introduction

Sepsis results from the presence of systemic or localised infections, initiating devastating proinflammatory effects on the microcirculation [1-3]. These effects lead to increased microvascular permeability and leukocyte activation, along with reduced vascular resistance and blood flow [4], and without treatment they may result in tissue damage, multiple organ failure and death. Severe sepsis has a mortality rate of approximately 30% in the USA [5], which represents the second largest killer after cardiovascular disorders. Corticosteroids and drotrecogin-alfa have been recommended for use in some circumstances [6], but effective therapeutic options remain scarce and basic treatment still revolves around optimising fluid resuscitation and antibiotic therapy. In addition, these therapies may improve tissue perfusion but do not reverse active inflammatory processes, thus there is currently a great need for novel therapeutic strategies for severe sepsis.

Angiopoietin (Ang-1) is an oligomeric-secreted glycoprotein that comprises, together with Ang-2, Ang-3 and Ang-4, a vascular-specific family of growth factors [7]. These ligands bind to the Tie-2 receptor, which is expressed on the vascular endothelium. Ang-1 is essential during angiogenesis, being required for correct organisation and maturation of newly formed vessels [7]. However, it also maintains the structural integrity of the mature vasculature [8], regulating microvascular permeability via several mechanisms, including: (i) Ang-1-bound Tie-2 signalling through Akt to endothelial nitric oxide synthase (eNOS), promoting quiescence and survival; (ii) sequestration of non-receptor tyrosine kinase Src through the RhoA downstream target mDia, which prevents Src-mediated vascular endothelial (VE)-cadherin internalisation, thus stabilising inter-endothelial junctions [8]. Being part of the intracellular pathway for nitric oxide (NO)-mediated vasodilation, eNOS also modulates blood flow and tissue perfusion [9].

Use of Ang-1 has proven beneficial in murine models of endotoxemia, as adenoviral-delivered Ang-1 improved survival rates from 60% to 91% 60 hours following lipopolysaccharide (LPS) administration, correlating with reduced lung oedema and injury [10]. More recently, human recombinant Ang-1, administered 8 hours before and in combination with LPS, also protected mice against pulmonary hyperpermeability *in vivo *through a p190 RhoGAP-dependent mechanism [11]. However, adenoviral delivery is not plausible in humans, while a large-scale production of recombinant Ang-1, which has a short half-life *in vivo *[12], is hindered by the aggregation and insolubility of this protein [13]. Moreover, septic patients need efficacious treatments after the disease has already developed [6].

With Ang-1 demonstrating exciting therapeutic potential during sepsis, we considered it important to find alternatives that acted via the Tie-2 pathway with potential for translation into human medicine. Matrilin-1-angiopoietin-1 (MAT.Ang-1) is one such compound, being more soluble (> 95% vs. 60 to 70%) than the native Ang-1. We produced this Ang-1 variant by replacing the central coiled-coil domain and N-terminal domain of Ang-1 with the short coiled-coil domain of human matrilin-1 [14]. In our study, we used MAT.Ang-1 rather than the more well-known variant COMP.Ang-1, as MAT.Ang1 has comparable activity with wildtype Ang-1, similarly forming a mixture of tetramer and trimer units [13]. The comparable activity between Ang-1 and MAT.Ang-1 also suggests that these basic units are active species.

This study investigated the protective effects against sepsis by MAT.Ang-1 *in vivo*, focusing on changes in microvascular permeability, resistance and blood flow. Importantly, we used a therapeutic protocol where MAT-Ang-1 was administered 20 hours after the onset of sepsis. We then determined whether any acute beneficial effects by MAT-Ang-1 were associated with concurrent modulation of inflammatory or angiogenesis factor expression, and the eNOS-Akt or VE-cadherin signalling pathways.

## Materials and methods

### Animals

Male C3H/HeN mice (7 to 10 weeks old; *n *= 24) were obtained from Charles River (Margate, Kent; UK). All procedures were performed in compliance with the UK Home Office Animal Scientific Procedures Act (1986), under HO project licence number 40/2972, with rigorous ethical and statistical review by both the Home Office and the University of Sheffield. Investigations conformed to the Guide for the Care and Use of Laboratory Animals published by the US National Institutes of Health (NIH Publication No. 85-23, revised 1996; Assurance No. A5463-01 for the University of Sheffield).

### MAT.Ang-1 production

The fibrinogen-like domain of human Ang1 (UniProt Q15389, residues 266 to 498), fused at its N terminus to the coiled-coil domain of human matrillin-1 (UniProt P21941, residues 445 to 496, Mat1-Ang1), was amplified by PCR and cloned into a modified pCEP-Pu vector that contained a secretory signal sequence for BM-40/osteonectin [15]. The recombinant protein was obtained by expression in HEK293 EBNA cells using Lipofectamine according to the manufacturer's instructions (Invitrogen; Paisley, UK). After selection with puromycin (5 μg/ml), cells were expanded for the production of the recombinant protein. Serum-free supernatants were harvested from transfected cells, and the recombinant protein was purified by immobilised metal affinity chromatography on Ni^2+^-Sepharose according to the manufacturer's instructions (Amersham Biosciences; Little Chalfont, Buckinghamshire; UK), dialysed against PBS and stored at -80°C for further use.

### Dorsal microcirculatory chamber

Mice were anaesthetised with hypnorm and diazepam (1:1, 0.1 ml/100 g intraperitoneally (i.p.)) and aluminium frames were surgically implanted onto the dorsal skinfold of animals. Briefly, on the left side a circular area of dermis and subcutis was surgically removed and the single layer of exposed striated muscle was covered by a glass window (8 mm diameter) [16]. All chambers were devoid of air bubbles, infection or vascular thrombosis.

### Intravital microscopy and experimental groups

Observation of the dorsal microcirculatory chamber was performed using intravital microscopy 3 days after surgery, during sedation with ketamine and xylazine (10 and 1 mg/kg i.p.).

Animals were placed on the stage of a modified Leica microscope (307-072.057; Leica; Milton Keynes, Buckinghamshire; UK), equipped with a tungsten lamp for transmitted light and a mercury lamp for fluorescent light microscopy (excitation filter 460 to 490 nm), and preparations were observed through a 10× objective (0.3 numerical aperture; Nikon; Kingston Upon Thames, Surrey; UK). Digital images were captured using a CCD camera (TK-C1360B; JVC; London, UK), displayed on a high-resolution monitor (PVM-1443; Sony; Thatcham, Berkshire; UK) and recorded by video data recorder (VDR-3000; Holdan; Hadfield, Glossop; UK) onto DVD disks (Verbatim 16× DVD+R; Verbatim; Egham, Surrey; UK) for later off-line analysis. The preparations were briefly scanned with low-level transmitted light for the study of the skeletal muscle microcirculation. Two areas of interest were identified - each containing main feeding arterioles (50 to 100 μm) and venules (100 to 250 μm), pre-capillary arterioles (20 to 30 μm) and post-capillary venules (30 to 45 μm) - and were recorded for 30 seconds using epi-illumination every hour between 20 and 24 hours.

Fluorescein isothiocyanate was bound to bovine serum albumin [17] and administered into the tail vein at 20 hours of the experimental protocol (66 kDa FITC-BSA, 200 μl/100 g intravenously; Sigma; Gillingham, Dorset; UK) to allow observation of the microcirculation. Animals were allocated into four experimental groups: control (*n *= 6), 1 mg/ml saline i.p. repeated at 0 and 19 hours; LPS (*n *= 6), B55:055, 600,000 endotoxin units per mg (Sigma), 1 mg/ml i.p. at 0 and 19 hours; LPS + MAT.Ang-1 (*n *= 6), 1 mg/ml LPS i.p. at 0 and 19 hours + 33 μg intravenously MAT.Ang-1 at 20 hours; and MAT.Ang-1 (*n *= 6), 33 μg intravenously at 20 hours [18,19].

### Image analysis: macromolecular leak and vessel diameter

The image analysis software Image-Pro Plus (version 6.0; Media Cybernetics; Rockville, MD; USA) was used off-line on fluorescent images obtained between 20 and 24 hours to measure interstitial fluorescence of FITC-BSA (macromolecular leak) adjacent to post-capillary venules [20]. Image-Pro Plus software assigned an integer value to the brightness of fluorescence, using an arbitrary 8-bit greyscale (range 0 to 255), at three distinct interstitial areas (900 μm^2^) immediately adjacent (< 2 mm) to a randomly selected post-capillary venule. This provided a mean value for the leakage from one post-capillary venule per each animal at every time point.

To determine diameters of arterioles and venules, Image-Pro Plus was calibrated with a micrometer specifically designed for the camera and monitor. Three lines were drawn across the vessels (including the lumen and vessel wall) to obtain a median value (in micrometres), and the diameter of one randomly selected feeding arteriole, one feeding venule, one pre-capillary arteriole and one post-capillary venule were measured in each animal at every time point.

### Laser Doppler fluximetry: blood flow

At the end of the microscopic evaluation (24 hours of the experimental protocol), tissues in the chambers were scanned using a MoorLDI2 V5.x Doppler imager (Moor Instruments; Axminster, Devon; UK). This instrument used a single 2 mW, 632.8 nm visible red helium neon laser and was positioned 20 cm from the window chamber and scanned with a raster pattern over a 5 cm × 5 cm area inclusive of the microcirculation and surrounding tissue, with a spatial resolution of 256 × 256 pixels and a scan rate of 4 milliseconds/pixel. The laser beam was positioned perpendicular to the chamber and generated two-dimensional colour-coded images of blood flow with corresponding photo-images based on light intensity. Images were stored on a compatible laptop computer. Perfusion images were analysed using the MoorLDI dedicated image analysis software (version 3.0). A square region of interest that included the main branch of the microcirculatory network was outlined on each image and used to calculate the area-averaged flux. All individual measurement values represent a mean flux from the measured region of interest in perfusion units.

### Cytokine and angiogenesis profiling

Twenty-four hours after LPS administration, abdominal muscle was removed and snap frozen. Tissues were homogenised in a lysis buffer containing PBS, Triton X-100 1% and protease inhibitor cocktail (Roche; Burgess Hill, West Sussex; UK), and their protein concentration was measured by the Bradford assay using BSA as standard. Protein expression of cytokines/chemokines and angiogenic factors was determined by two different multiplex arrays for simultaneous detection (R&D Systems; Abingdon, Oxfordshire; UK) according to the manufacturer's protocol. A 200 μg sample of tissue proteins was used for this array.

### Immunoblotting

Proteins (20 or 80 μg; obtained as described above) were boiled (10 minutes) and loaded on 12% SDS-PAGE and transferred onto nitrocellulose membranes for 45 minutes at 250 mA. Nonspecific antibody binding to the membrane was blocked with 5% nonfat milk in PBS-Tween 20 (0.1%, v/v) for 1 hour at room temperature. Membranes were then incubated overnight at 4°C with the primary antibodies used against eNOS (1:1,000; BD Biosciences; Oxford, Oxfordshire; UK), phospho-eNOS (1:200; Cell Signaling; Danvers, MA; USA), inducible nitric oxide synthase (iNOS, 1:200; Santacruz; Santacruz, CA; UK), Tie-2 (1:2,000; R&D), phospho-Tie-2 (1:200; R&D), VE-cadherin (1:200; Cayman; Ann Arbor, MI;USA), phospho-VE-cadherin Y658 (1:1,000; Abcam; Cambridge, Cambridgeshire; UK), Akt (1:5,000; R&D), phospho-Akt S473 (1:2,000; R&D), and Ang-2 (1:1,000; Alpha Diagnostic; San Antonio, TX; USA). Detection blots were washed with PBS-Tween 20 (0.1% v/v) at 10-minute intervals for 50 minutes and were incubated with horseradish peroxidase-anti-rabbit, horseradish peroxidase-anti-mouse or horseradish peroxidase-anti-goat IgG (1:1,000 to 1:2,000) for 1 hour at room temperature. Primary and secondary antibodies were dissolved in PBS-Tween 20 0.1% v/v containing 5% nonfat milk. Membranes were then washed with PBS-Tween 20 (0.1% v/v) at 10-minute intervals for 40 minutes and the immunoreactive bands were visualised using an enhanced chemiluminescence system. Every blot was stripped and reprobed for actin (1:1,000; Sigma) as internal control.

### Serum nitric oxide assay

Twenty-four hours after LPS administration, blood serum was obtained by cardiac puncture and centrifuged at 13,000 relative centrifugal force (rcf) for 5 minutes. The Stressgen Nitric Oxide (total) Detection Kit, which quantifies total nitrite colorimetrically using the Griess reaction, was used according to the manufacturer's instructions (Stressgen Biotechnologies; Victoria, British Columbia; Canada). Frozen serum was defrosted, diluted 1:10, and analysed in duplicate. The absorbance in each well between 540 and 570 nm was determined using a plate reader. The average net absorbance for each standard and sample was calculated by subtracting the average zero standard absorbance from the average absorbance for each standard and sample. This allowed nitrite levels to be calculated as an indicator of total NO production.

### Statistical analysis

All data were expressed as the mean ± standard error of the mean and analysed using Graphpad Prism version 5.0 (Graphpad Software; San Diego, CA; USA). Two-way analysis of variance followed by the Bonferroni *post-hoc *test to identify points of significance or one-way analysis of variance followed by appropriate *post-hoc *test or Student's *t *test were used as appropriate. *P *< 0.05 was considered significant.

## Results

### MAT.Ang-1 protects mice against LPS-induced microvascular hyperpermeability

**Figure 1:**
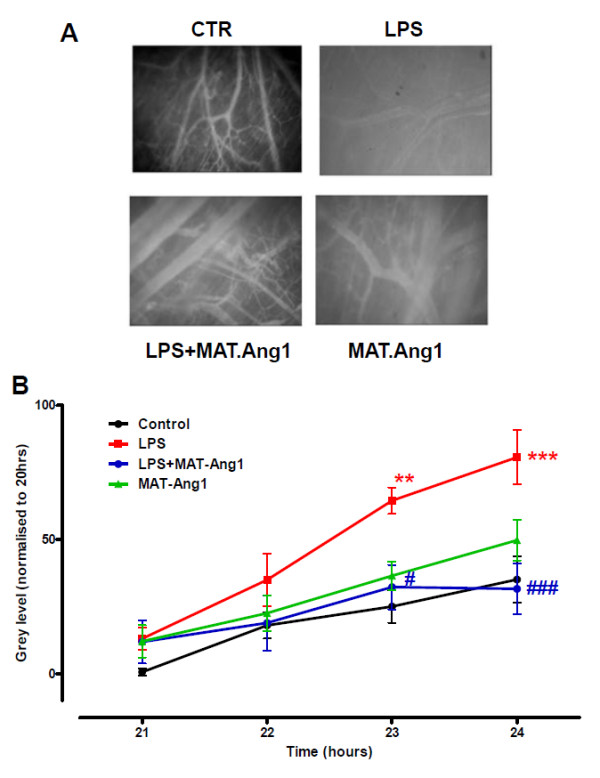
**MAT.Ang-1 reduces lipopolysaccharide-induced microvascular leak**. (**A**) Representative images at 24 hours of murine skeletal muscle microvasculature from control (CTR), lipopolysaccharide (LPS)-treated, LPS + matrilin-1-angiopoietin-1 (MAT-Ang1)-treated and MAT-Ang1-treated groups. (**B**) Data expressed as mean ± standard error of the mean for change in grey level indicating macromolecular leak from 20 hours (*n *= 6). **P < 0.01 and ***P < 0.001 vs. control. ^#^*P *< 0.05 and ^###^*P *< 0.001 vs. LPS.

LPS caused a significant increase in macromolecular leak (normalised grey level) compared with controls (LPS group vs. controls, 64.5 ± 4.9 vs. 25.0 ± 6.1 at 23 hours, *P *< 0.01; 80.7 ± 10.1 vs. 35.1 ± 8.6 at 24 hours, *P *< 0.001). MAT.Ang-1 reduced LPS-induced leak at 23 hours (32.2 ± 8.3, *P *< 0.05) and at 24 hours (31.6 ± 9.5, *P *< 0.001) (Figure 1). MAT.Ang-1 alone did not significantly increase macromolecular leak compared with controls (36.5 ± 5.2 at 23 hours and 49.7 ± 7.5 at 24 hours). However, vessel diameters of arterioles and venules were not significantly different among any experimental group (Table S1 and Figures S1, S2 in Additional file 1).

### Microvascular blood flow in sepsis is improved by MAT.Ang-1

**Figure 2:**
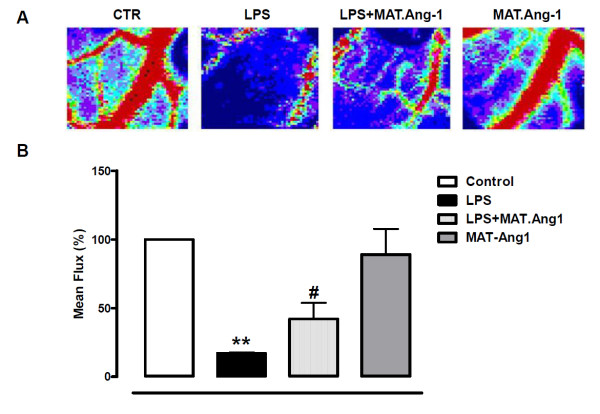
**MAT.Ang-1 ameliorates microvascular blood flow in sepsis**. (**A**) Representative images from Doppler scanning at 24 hours of control (CTR), lipopolysaccharide (LPS), LPS + matrilin-1-angiopoietin-1 (MAT-Ang1) and MAT-Ang1 groups. Colours correspond to a perfusion range from 0 (black; lowest flux) to 1,000 (red; highest flux). (**B**) Data expressed as mean ± standard error of the mean of flux calculated as a percentage of control (*n *= 5). **P < 0.01 vs. control. ^#^*P *< 0.05 vs. LPS.

In response to LPS, mean perfusion (percentage of control) decreased to 17.1 ± 0.6% at 24 hours (*P *< 0.01) (Figure 2); whereas in the presence of LPS, MAT.Ang-1 increased mean perfusion to 42.1 ± 11.7% (*P *< 0.05). However, mean perfusion in response to MAT.Ang-1 alone (89.0 ± 18.7%) was not different from controls.

### MAT.Ang-1 reduces the inflammatory response during endotoxemia

**Figure 3:**
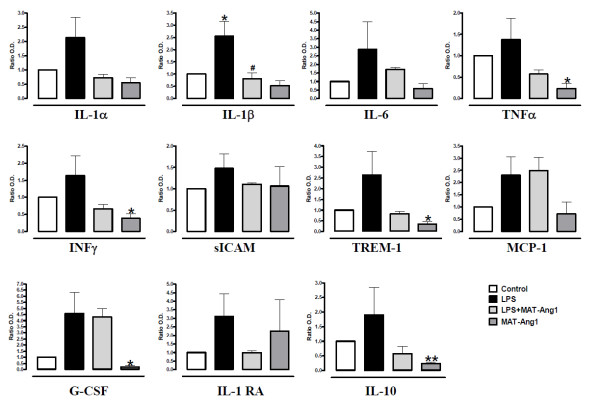
**MAT.Ang-1 reduces the inflammatory response in sepsis**. Cytokine, chemokine and growth factor levels detected at 24 hours in skeletal muscle from control, lipopolysaccharide (LPS)-treated, LPS + matrilin-1-angiopoietin-1 (MAT-Ang1)-treated and MAT.Ang1-treated groups. Data expressed as mean ± standard error of the mean of fold-change from control (*n *= 3 different experiments performed in duplicate). *P < 0.05, **P < 0.01 vs. control. ^#^*P *< 0.05 vs. LPS. G-CSF, granulocyte colony-stimulating factor; IL-1RA, IL-1 receptor antagonist; MCP-1, monocyte chemotactic protein-1; sICAM, soluble intracellular adhesion molecule-1; TREM-1, triggering receptor expressed on myeloid cells-1.

LPS induced a trend of increased expression for the majority of cytokines analysed in skeletal muscle at 24 hours (Table S2 in Additional file 1), including the proinflammatory cytokines IL-1α, IL-1β, IL-6, TNFα, IFNγ, soluble intracellular adhesion molecule-1 (sICAM-1) and triggering receptor expressed on myeloid cells-1 (TREM-1), which were reduced by post-treatment with MAT.Ang-1 (Figure 3). In contrast, monocyte chemotactic protein-1 (MCP-1) and granulocyte colony-stimulating factor (G-CSF) were increased by LPS, but not modulated by MAT.Ang-1. Notably, LPS-induced increased IL-1β expression was significantly reduced by post-treatment with MAT.Ang-1 (*P *< 0.05) (Figure 3). MAT.Ang-1 alone also reduced the expression of TNFα, IFNγ, triggering receptor expressed on myeloid cells-1 and G-CSF (*P *< 0.05). Furthermore, the anti-inflammatory cytokines IL-10 and IL-1 receptor antagonist exhibited increased expression during endotoxemia, but were reduced by post-treatment with MAT.Ang-1. IL-10 was also reduced by MAT-Ang-1 alone (*P *< 0.01) (Figure 3).

### Angiogenic markers are reduced in sepsis and not induced by MAT.Ang-1

**Figure 4:**
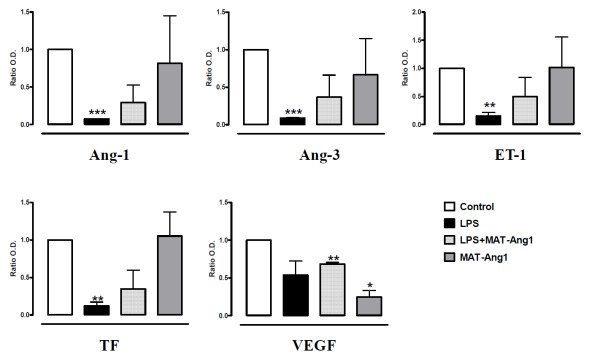
**Angiogenic factors are not induced by MAT.Ang-1**. Angiogenic markers detected at 24 hours in skeletal muscle from control, lipopolysaccharide (LPS), LPS + matrilin-1-angiopoietin-1 (MAT-Ang1) and MAT.Ang1 groups. Data are mean ± standard error of the mean of fold-change from control (*n *= 3 different experiments performed in duplicate). *P < 0.05, **P < 0.01 and ***P < 0.001 vs. control. Ang, angiopoietin; ET-1, endothelin-1; TF, tissue factor; VEGF, vascular endothelial growth factor.

The majority of angiogenic factors were significantly reduced at 24 hours in response to LPS (Table S3 in Additional File 1). In particular, Ang-1 and Ang-3 (*P *< 0.001), endothelin-1 (*P *< 0.01) and tissue factor (*P *< 0.01) were significantly reduced (Figure 4). Post-treatment with MAT.Ang-1 during endotoxemia did not increase significantly the expression of Ang-1, Ang-3, endothelin-1 and tissue factor. In addition, vascular endothelial growth factor (VEGF) expression was reduced in the presence of MAT.Ang-1 alone (*P *< 0.05) and during endotoxemia (*P *< 0.01).

**Figure 5:**
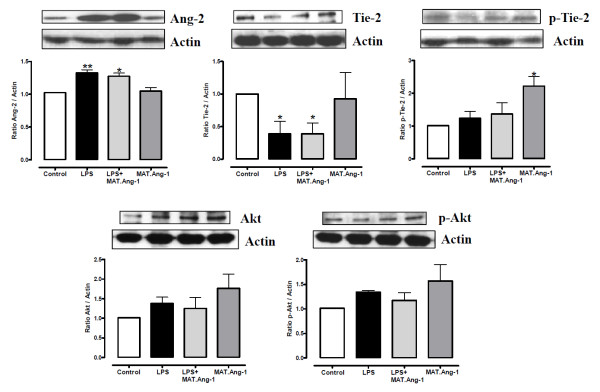
**Western blot analysis of angiopoietin-2, Tie-2 receptor and Akt**. Detection of angiopoietin (Ang)-2, Tie-2, phospho-Tie-2, Akt and phospho-Akt by immunoblotting at 24 hours in skeletal muscle from control, lipopolysaccharide (LPS)-treated, LPS + matrilin-1-angiopoietin-1 (MAT-Ang1)-treated and MAT.Ang1-treated groups. Blots shown are representative of four animals per experimental group. Data on graphs expressed as mean ± standard error of the mean of fold-change from control (*n *= 4). *P < 0.05 and **P < 0.01 vs. control.

Ang-2 expression was significantly increased in endotoxemia at 24 hours (*P *< 0.01), and was unchanged by MAT.Ang-1 administration (Figure 5). Conversely, Tie-2 receptor expression was significantly reduced by LPS at 24 hours (*P *< 0.05), and was unchanged by MAT.Ang-1 (Figure 5). Furthermore, Tie-2 phosphorylation at Y1100 by MAT.Ang-1 was observed in normality but not in response to LPS (Figure 5). At the same time point, the signalling survival protein Akt and its phosphorylation at S473 were not significantly different amongst the experimental groups (Figure 5).

### MAT.Ang-1 increases VE-cadherin phosphorylation in sepsis

**Figure 6:**
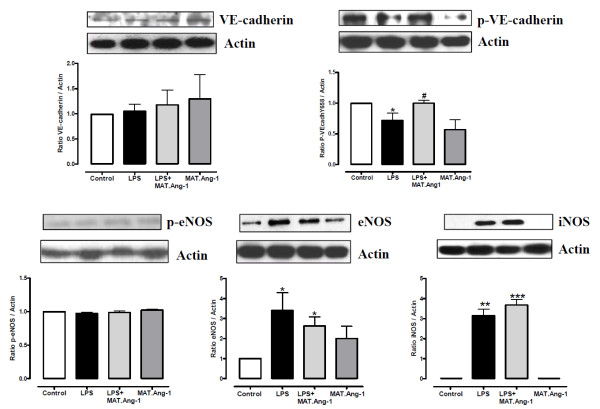
**Western blot analysis of vascular endothelial-cadherin, endothelial nitric oxide synthase and inducible nitric oxide synthase**. Detection of vascular endothelial (VE)-cadherin, phospho-VE-cadherin, phospho-endothelial nitric oxide synthase (phospho-eNOS), eNOS and inducible nitric oxide synthase (iNOS) by western blot analysis at 24 hours in skeletal muscle from control, lipopolysaccharide (LPS)-treated, LPS + matrilin-1-angiopoietin-1 (MAT-Ang1)-treated and MAT.Ang1-treated groups. Blots shown are representative of four animals per experimental group. Data on graphs expressed as mean ± standard error of the mean of fold-change from control (*n *= 4). *P < 0.05, **P < 0.01, ***P <0.001 vs. control. ^#^*P *< 0.05 vs. LPS.

No change in VE-cadherin expression was observed amongst the experimental groups (Figure 6). Nevertheless, during endotoxemia VE-cadherin phosphorylation at Y658 was increased in MAT.Ang-1-treated animals compared with those treated with LPS alone (*P *< 0.05) (Figure 6). MAT.Ang-1 alone also reduced Y658 phosphorylation, but this did not reach significance (*P *= 0.07).

### LPS-induced increase in nitric oxide synthase expression is not changed by MAT.Ang-1

eNOS phosphorylation at Ser1177 did not vary in skeletal muscle amongst the experimental groups (Figure 6). Nevertheless, eNOS protein expression increased after 24 hours of endotoxemia and this was not altered by post-treatment with MAT.Ang-1 (*P *< 0.05 vs. control) (Figure 6). iNOS expression was neither detected in controls nor in the presence of MAT.Ang-1 alone, and the LPS-induced increase in iNOS expression (*P *< 0.01) was unchanged by MAT.Ang-1 (Figure 6).

Serum NO, measured as total nitrite levels, increased from 53.2 ± 26.6 μmol/l in controls to 342.1 ± 118.7 μmol/l in endotoxemic mice (*P *< 0.05), and remained elevated with concurrent LPS and MAT.Ang-1 administration (551.2 ± 245.3 μmol/l). NO levels were also similar to controls with MAT.Ang-1 alone (52.7 ± 44.8 μmol/l).

## Discussion

This is the first *in vivo *demonstration that MAT.Ang-1, administered after the onset of sub-lethal endotoxemia, reduces LPS-induced macromolecular leak and improves microcirculatory blood flow in sepsis without changing vascular resistance. In contrast, MAT.Ang-1 alone does not induce leak or change microvascular flow and/or resistance in skeletal muscle under physiological conditions. Endotoxemia is locally associated with increased inflammation and reduced angiogenic factors. MAT.Ang-1 post-treatment reduces the majority of inflammatory mediators, including IL-1β, without increasing the expression of angiogenic factors. Under physiological conditions, MAT.Ang-1 does not induce local angiogenic markers, but reduces VEGF expression. Our data therefore show that MAT.Ang-1 protects the microcirculation from sepsis-induced vascular dysfunction and local inflammation without causing angiogenesis.

In search of an efficacious treatment for sepsis our study has focused on the microcirculation, based on recent evidence that microcirculatory dysfunction is a major feature of sepsis [2]. Microvascular dysfunction in sepsis is characterised by disruption of endothelial barrier function [21], but no effective pharmacological therapy is currently available to reduce the increased vascular permeability occurring in sepsis [22]. In our study, MAT.Ang-1 administered following the onset of sepsis protected the microcirculation against the barrier breakdown induced by experimental endotoxemia. Consistently, microcirculatory perfusion was found to be dramatically reduced in endotoxemic mice and improved following MAT.Ang-1 administration. Moreover, we applied the laser Doppler imaging technique to the window chamber model for the first time, and propose that the intravital microscopy-Doppler combination is a powerful technology to study the microcirculation in health and disease. Notably, MAT.Ang-1 did not change the arteriolar diameter during sepsis and normal conditions, suggesting that: the effects of MAT.Ang-1 on microvascular permeability and blood flow in pathological (endotoxemia) and physiological conditions are not dependent on changes in microvascular resistance; and MAT.Ang-1-induced recovery of microcirculatory tissue perfusion during sepsis is due to preservation of endothelial barrier integrity.

Endothelial cell damage and inflammation have been considered the basis of increased vascular permeability and altered blood flow [23]. We used a protein antibody array for the simultaneous detection of 40 different cytokines, chemokines and growth factors to investigate whether MAT.Ang-1 protected the microcirculation by modulating the inflammatory response occurring in sepsis. Most inflammatory cytokines showed a trend of increased expression in endotoxemia and reduction in the presence of MAT.Ang-1. Notably, the reduced expression of IL-1β by MAT.Ang-1 may account for the beneficial action on blood flow and tissue perfusion in endotoxemia, as IL-1β induces neutrophil migration into tissue, causing microvascular stasis in sepsis [24,25]. Furthermore, TNFα and IL-6 are considered biomarkers of sepsis [26,27], and in this study their levels were reduced locally by MAT.Ang-1 during endotoxemia. By contrast, G-CSF and monocyte chemotactic protein-1 expression remained elevated after MAT.Ang-1 administration in sepsis. G-CSF has been included in clinical trials for the prophylaxis and treatment of sepsis [28,29], while monocyte chemotactic protein-1 may be involved in the pathophysiology of sepsis as plasma levels of this chemokine are elevated in septic patients [30], but its role remains controversial [31,32].

The role of angiogenic factors in the pathogenesis of sepsis is largely unexplored, but therapeutic induction of angiogenesis in damaged vessels has been suggested as a valid approach [33,34]. Our results demonstrate a significant reduction during endotoxemia in local angiogenic factors, including Ang-1, Ang-3, endothelin-1 and tissue factor, with no marked changes by MAT.Ang-1. Consistently, we observed no change in expression and phosphorylation of the pro-survival factor Akt or eNOS phosphorylation at Ser1177 at the same time point. The absence of pro-angiogenic effects by MAT.Ang-1 is unsurprising because Ang-1 regulates vascular maturation at later stages of the angiogenic cascade [8]. Moreover, our findings show that LPS increased Ang-2 protein levels with concomitant reduction in Tie-2 receptor expression in skeletal muscle, thus providing further *in vivo *evidence that endotoxemia triggers inhibition of the Ang/Tie system, which may contribute to vascular dysfunction in sepsis [35]. Furthermore, our results suggest that MAT.Ang-1 acts via Tie-2 *in vivo *with increased receptor phosphorylation, although specific receptor activation by MAT.Ang-1 *in vivo *is difficult to verify because concomitant receptor blockade - for example, by Ang-2 in inflammation - modulates vascular function (see also Figure S3 in Additional file 1). However, Tie-2phosphorylation was unchanged during endotoxemia.

Previous studies demonstrated that VE-cadherin phosphorylation at the critical tyrosine Y658 may be sufficient to inhibit cell barrier function [36]. Nevertheless, disassembly of VE-cadherin junctions has been shown to trigger an intracellular negative signal for limiting transendothelial leukocyte migration in mice 6 hours after challenge with LPS [37]. Increased phosphorylation at Y658 in endotoxemia by MAT.Ang-1, in addition to reduced IL-1β expression, is therefore likely to reduce neutrophil infiltration and microvascular stasis in sepsis.

During the progression of sepsis, proinflammatory cytokines and LPS stimulate NO production mainly through increased expression of iNOS, which has been implicated in the pathophysiology of microcirculatory failure and organ dysfunction [38]. However, in the present study neither iNOS nor total nitrite levels were modulated by the administration of MAT.Ang-1, despite the observed beneficial effects on reducing inflammation. Moreover, MAT.Ang-1 also sustained the increase in tissue eNOS expression during endotoxemia, which may act as a protective mechanism in sepsis by improving microcirculatory flow [39]. Recent evidence also suggests that high levels of NO can be beneficial in sepsis by improving tissue perfusion and oxygen extraction [40], thus NO is part of a complex mechanistic pathway and further studies are required before firm conclusions can be made regarding an inflammatory role for NO [41].

Previous studies have used COMP-Ang-1 prophylactically to prevent vascular inflammation and organ dysfunction during sepsis [42,43], and our findings demonstrate that MAT.Ang-1 has similar protective effects even administered after the onset of endotoxemia. Since MAT.Ang-1 and Ang-1 have similar physiological activity, this suggests that our novel variant is likely to demonstrate increased therapeutic potential when compared with COMP.Ang-1 [13], although this requires confirmation. Biological MAT.Ang-1 units consist of trimers and tetramers rather than more active pentamers, and hence - compared with COMP.Ang-1 - side effects such as venous malformations are less likely to occur when administered systemically [44]. Moreover, while previous studies have shown protective effects by repeated prophylactic or therapeutic injections of recombinant Ang-1 [45] or the Tie-2 agonist Vasculotide [46] in murine sepsis, the current study demonstrates that a single administration of the stable variant MAT.Ang-1 after the onset of sepsis improved endotoxemia-induced microvascular dysfunction.

Using the variant MAT.Ang-1, this *in vivo *study also further investigated the effects of acute activation of the angiopoietin system under physiological conditions. In skeletal muscle, TNFα, IFNγ, IL-10 and G-CSF expression were reduced by MAT.Ang-1, further confirming that Ang-1 may act as an endogenous vessel stabiliser [47]. By contrast, MAT.Ang-1 did not alter the expression of any angiogenic factors detected, suggesting that induction of angiogenesis is unlikely at the dose used. Interestingly, only VEGF expression was reduced by MAT.Ang-1 administration. Indeed, a functional competition has previously been described between VEGF and Ang-1, with Ang-1 preventing vascular leakage in response to VEGF administration [48]. VEGF also induces cleavage of Tie-2 receptors, creating soluble forms (soluble Tie-2) that sequester Ang-1 and inhibit ligand-mediated Tie-2 activation [49]. Our data suggest for the first time that a feedback mechanism exists by which Ang-1 downregulates VEGF-mediated vascular instability, resulting in vascular stabilisation.

## Conclusion

Our study provides evidence that the microcirculation is protected from sepsis-induced vascular dysfunction *in vivo *by acute parenteral administration of MAT.Ang-1, a novel variant of Ang-1, after the onset of endotoxemia. To our knowledge, no other studies have successfully used an angiopoietin variant to reverse inflammation - and our data show that vascular protection is mediated by effects on the inflammatory response in sepsis rather than stimulation of angiogenesis. Further studies are also warranted to examine the ability of MAT.Ang-1 to reduce mortality in more severe models of sepsis. Pharmacotherapeutic targeting of the angiopoietin system in sepsis, in addition to other cardiovascular disorders, is thus possible using chemical variants of Ang-1 that improve its pharmacokinetic and pharmacodynamic characteristics *in vivo*, allowing optimal delivery [50]. Improved microvascular blood flow, reduced inflammation and lack of activation of angiogensis by MAT.Ang-1 post-treatment in our nonlethal *in vivo *model of sepsis provide the basis for further clinical investigation with septic patients.

## Key messages

• Ang-1 showed therapeutic potential for the treatment of sepsis and cardiovascular disorders, but further pharmacological characterisation and drug delivery is required. This study aimed to investigate the acute effects of the stable and active Ang-1 variant MAT.Ang-1 on the microcirculatory function (permeability, resistance and blood flow) in an experimental model of sepsis.

• Endotoxemia resulted in microvascular leak and reduction of tissue perfusion, which was ameliorated by MAT.Ang-1 post-treatment.

• MAT.Ang-1 post-treatment reduced the inflammatory response by LPS but did not significantly induce the expression of angiogenic factors.

• MAT.Ang-1 alone did not induce leak or increase angiogenic factors but, in contrast, reduced VEGF expression in controls.

• Administration of MAT.Ang-1 after the onset of sepsis protects the microcirculation from endotoxemia-induced vascular dysfunction by reducing the inflammatory response but with no concomitant induction of angiogenic factors, thus representing a novel drug for the treatment of sepsis.

## Abbreviations

Akt: RAC - a serine/threonine-protein kinase; Ang: angiopoietin; BSA: bovine serum albumin; eNOS: endothelial nitric oxide synthase; G-CSF: granulocyte colony-stimulating factor; IL: interleukin; iNOS: inducible nitric oxide synthase; i.p.: intraperitoneally; LPS: lipopolysaccharide; MAT.Ang-1: matrilin-1-angiopoietin-1; mDia: mammalian diaphanous; NO: nitric oxide; PBS: phosphate-buffered saline; PCR: polymerase chain reaction; Tie-2: tyrosine kinase with immunoglobulin-like and endothelial growth factor-like domains 2; VE: vascular endothelial; VEGF: vascular endothelial growth factor.

## Competing interests

The authors declare that they have no competing interests.

## Authors' contributions

All *in vivo *and *ex vivo *experiments in this study were performed by AA. MAT.Ang-1 was produced by MT and PP in collaboration with RAK. JJW performed preliminary experiments in collaboration with KR. ZLB and NJB had the initial idea, obtained the funding, led and supervised the project. The manuscript was written by AA and ZLB in consultation with NJB and RAK, who contributed to aspects of data analysis. All authors read and approved the final version of this manuscript.

## Supplementary Material

Additional file 1 **Table S1 presenting vessel diameters from control, LPS, LPS+MAT-Ang1 and MAT-Ang1 groups**. Table S2 presenting protein expression of cytokines and chemokines in skeletal muscle from control, LPS, LPS+MAT-Ang1 and MAT-Ang1 groups. Table S3 presenting protein expression of angiogenic factors in skeletal muscle from control, LPS, LPS+MAT-Ang1 and MAT-Ang1 groups. Figure S1 showing diameters of primary arterioles and venules from control, LPS, LPS+MAT-Ang1 and MAT-Ang1 groups. Figure S2 showing diameters of pre-capillary arterioles and post-capillary venules from control, LPS, LPS+MAT-Ang1 and MAT-Ang1 groups. Figure S3 showing the effect of Tie-2 receptor antagonist NLLMAAS on macromolecular leak. Click here for file
