# Supplementary material for: Angiopoietin-1 variant reduces LPS-induced microvascular dysfunction in a murine model of sepsis
Source: Crit Care. 2012 Oct 4;16(5):R182. doi: 10.1186/cc11666 (PMC3682284; doi:10.1186/cc11666)
Supplement: Additional file 1 — Table S1 presenting vessel diameters from control, LPS, LPS+MAT-Ang1 and MAT-Ang1 groups. Table S2 presenting protein expression of cytokines and chemokines in skeletal muscle from control, LPS, LPS+MAT-Ang1 and MAT-Ang1 groups. Table S3 presenting protein expression of angiogenic factors in skeletal muscle from control, LPS, LPS+MAT-Ang1 and MAT-Ang1 groups. Figure S1 showing diameters of primary arterioles and venules from control, LPS, LPS+MAT-Ang1 and MAT-Ang1 groups. Figure S2 showing diameters of pre-capillary arterioles and post-capillary venules from control, LPS, LPS+MAT-Ang1 and MAT-Ang1 groups. Figure S3 showing the effect of Tie-2 receptor antagonist NLLMAAS on macromolecular leak. [file cc11666-S1.DOCX]

**SupplementaRY DATA**

*Vessel diameter*

A trend of vasoconstriction was observed in primary (feeding) arterioles from LPS-injected mice (Supplemental Table 1 and Supplemental Figure 1A), but this did not reach significance (p=0.15 at 24 hours). MAT.Ang-1 administration (i.v.) did not induce any change of the arteriolar diameter over 4 hours, either alone or during endotoxemia (Supplemental Table 1 and Supplemental Figure 1A). On the contrary, a trend of dilation of primary venules was observed in LPS-injected mice (Supplemental Table 1 and Supplemental Figure 1B), but this also did not reach significance (p=0.06 at 24 hours). MAT.Ang-1 administration did not induce any change of the venular diameter either alone or during endotoxemia (Supplemental Table 1 and Figure 1B).

Similar trends were observed in pre-capillary arterioles and post-capillary venules (Supplemental Table 1 and Supplemental Figure 2A and B), with vasoconstriction and vasodilation of arterioles (Supplemental Table 1 and Figure 2A) and venules (Supplemental Table 1 and Figure 2B) respectively in LPS-injected mice. No significant change in the arteriolar or venular diameter was induced by MAT.Ang-1, either alone or during endotoxemia (Supplemental Table 1 and Supplemental Figure 2A and B).

**Supplemental Table 1:** Vessels diameters (µm).

|  | **20 hours** | **21 hours** | **22 hours** | **23 hours** | **24 hours** |
| --- | --- | --- | --- | --- | --- |
| ***Primary arterioles*** | | | | | |
| **Control** | 86.6±15.5 | 94.5±11.9 | 88.9±13.4 | 77.2±11.4 | 78.1±12.7 |
| **LPS** | 82.6±11.9 | 78.8±9.5 | 73.1±7.8 | 70.8±4.6 | 62.0±8.0 |
| **LPS+MAT.Ang-1** | 78.4±9.1 | 71.5±7.8 | 68.4±7.3 | 68.2±9.3 | 59.8±9.3 |
| **MAT.Ang-1** | 96.2±8.8 | 87.4±11.3 | 90.1±11.3 | 86.9±11.4 | 79.9±11.9 |
| ***Primary venules*** | | | | | |
| **Control** | 159.1±21.2 | 167.0±25.3 | 182.3±24.3 | 175.0±27.4 | 167.9±22.8 |
| **LPS** | 238.0±27.4 | 256.0±27.5 | 239.0±21.9 | 240.3±27.7 | 232.7±29.6 |
| **LPS+MAT.Ang-1** | 241.2±23.2 | 253.1±22.9 | 256.4±22.0 | 260.4±19.9 | 269.9±18.6 |
| **MAT.Ang-1** | 140.7±15.2 | 142.2±16.0 | 137.3±12.9 | 148.8±17.3 | 147.8±17.4 |
| ***Pre-capillary arterioles*** | | | | | |
| **Control** | 25.3±4.5 | 28.8±3.0 | 27.1±3.0 | 28.5±2.4 | 29.6±4.0 |
| **LPS** | 27.4±3.4 | 27.7±3.6 | 26.5±3.4 | 23.4±1.9 | 24.6±1.2 |
| **LPS+MAT.Ang-1** | 24.7±3.1 | 24.7±3.1 | 24.8±1.5 | 24.2±1.4 | 21.9±0.9 |
| **MAT.Ang-1** | 24.2±2.9 | 25.6±3.1 | 26.7±1.5 | 27.7±4.2 | 24.9±3.0 |
| ***Post-capillary venules*** | | | | | |
| **Control** | 31.9±3.4 | 32.7±3.2 | 33.4±2.8 | 34.1±2.9 | 33.9±2.9 |
| **LPS** | 34.3±3.4 | 37.1±4.0 | 39.5±3.5 | 36.0±2.6 | 39.9±2.1 |
| **LPS+MAT.Ang-1** | 33.5±3.3 | 32.4±2.8 | 31.7±4.1 | 35.7±2.6 | 37.6±1.3 |
| **MAT.Ang-1** | 33.2±1.9 | 34.6±2.7 | 31.8±2.6 | 42.7±3.9 | 37.3±3.8 |

Data are mean±SEM; n=6 per each experimental group.

**A B**

**Supplemental Figure 1:** Diameter of primary arterioles (A) and venules (B) in control, LPS, LPS+MAT-Ang1 and MAT-Ang1 group. Data are mean±SEM; n=6 per each experimental group.

**A B**

**Supplemental Figure 2:** Diameter of pre-capillary arterioles (A) and post-capillary venules (B) in control, LPS, LPS+MAT-Ang1 and MAT-Ang1 group. Data are mean±SEM; n=6 per each experimental group.

**Supplemental Table 2:** Cytokines and chemokines in skeletal muscle (data are expressed as fold change to control).

|  | **LPS** | **LPS+MAT.ANG-1** | **MAT.ANG-1** |
| --- | --- | --- | --- |
| **Pro-inflammatory cytokines** | | | |
| TNF-α | 1.378±0.498 | 0.572±0.090* | 0.232±0.123* |
| IL-1α | 2.132±0.707 | 0.728±0.126 | 0.546±0.186 |
| IL-1β | 2.543±0.619* | 0.817±0.236^#^ | 0.525±0.199 |
| IL-2 | 0.817±0.243 | 0.711±0.123 | 0.777±0.256 |
| IL-5 | 2.061±0.894 | 0.755±0.063 | 0.306±0.128* |
| IL-6 | 2.873±1.621 | 1.710±0.112* | 0.583±0.300 |
| IL-7 | 1.602±0.939 | 0.465±0.112* | 0.327±0.201 |
| IL-12(p70) | 3.746±1.610 | 0.836±0.359 | 0.399±0.146 |
| IL-13 | 1.931±1.048 | 0.523±0.149 | 0.324±0.155* |
| IL-16 | 2.283±0.624 | 0.888±0.339 | 1.304±0.731 |
| IL-17 | 1.167±0.051 | 0.682±0.116^#^ | 0.477±0.176 |
| IL-23 | 0.881±0.189 | 0.719±0.120 | 0.652±0.265 |
| IL-27 | 0.769±0.309 | 0.722±0.171 | 0.403±0.199 |
| IFN-γ | 1.633±0.585 | 0.663±0.133 | 0.382±0.129* |
| C5a | 0.861±0.313 | 0.646±0.116 | 0.463±0.181 |
| TREM-1 | 2.644±1.097 | 0.825±0.105 | 0.347±0.117* |
| RANTES | 1.149±0.359 | 0.846±0.023* | 0.462±0.170 |
| sICAM | 1.477±0.343 | 1.106±0.184 | 1.063±0.453 |
| **Anti-inflammatory cytokines** | | | |
| IL-1 Rec. Antagonist | 3.104±1.318 | 0.985±0.126 | 2.262±1.819 |
| IL-4 | 1.307±0.534 | 0.601±0.061* | 0.389±0.132* |
| IL-10 | 1.905±0.938 | 0.569±0.250 | 0.233±0.053** |
| TIMP-1 | 1.244±0.331 | 0.991±0.149 | 0.774±0.448 |
| **Chemokines** |  |  |  |
| MCP-1 | 2.306±0.752 | 2.486±0.545 | 0.715±0.486 |
| MCP-5 | 1.513±0.847 | 0.608±0.175 | 0.199±0.083* |
| MIP-1α | 4.881±2.597 | 1.424±0.272 | 0.371±0.115* |
| MIP-1β | 1.985±0.744 | 0.799±0.187 | 0.358±0.080* |
| MIP-2 | 1.431±0.439 | 1.943±0.237 | 0.569±0.218 |
| MIG | 2.521±1.250 | 2.088±0.796 | 0.310±0.158* |
| BLC | 1.151±0.227 | 0.711±0.172 | 0.453±0.298 |
| CCL-1 | 1.542±0.748 | 0.534±0.183 | 0.407±0.176 |
| Eotaxin | 1.667±0.481 | 0.730±0.345 | 0.333±0.085* |
| I-TAC | 1.617±0.690 | 0.643±0.121 | 0.314±0.108* |
| IP-10 | 2.443±1.046 | 1.500±0.194 | 0.333±0.193 |
| KC | 1.529±0.458 | 1.661±0.158 | 0.496±0.298 |
| SDF-1 | 0.950±0.475 | 1.092±0.460 | 0.411±0.241 |
| TARC | 2.333±0.811 | 0.876±0.186 | 0.363±0.217 |
| **Growth factors** |  |  |  |
| IL-3 | 1.587±0.599 | 0.765±0.160 | 0.345±0.186 |
| G-CSF | 4.601±1.707 | 4.302±0.677* | 0.206±0.100* |
| GM-CSF | 1.970±0.817 | 0.883±0.299 | 0.213±0.054** |
| M-CSF | 1.767±0.810 | 0.849±0.204 | 0.474±0.299 |

Values are means±SEM; n=3 per each experimental group. *p<0.05 and **p<0.01 vs. control; ^#^p<0.05 vs. LPS.

**Supplemental Table 3:** Angiogenic factors in skeletal muscle (data are expressed as fold change to control)

|  | **LPS** | **LPS+MAT.ANG-1** | **MAT.ANG-1** |
| --- | --- | --- | --- |
| ADAMTS1 | 0.229±0.082* | 0.406±0.310 | 1.554±0.880 |
| Amphiregulin | 0.281±0.051** | 0.446±0.277 | 2.298±2.026 |
| Angiogenin | 0.596±0.260 | 0.451±0.245 | 1.134±0.595 |
| Angiopoietin-1 | 0.077±0.002*** | 0.294±0.231 | 0.817±0.632 |
| Angiopoietin-3 | 0.089±0.005*** | 0.368±0.293 | 0.665±0.484 |
| Tissue Factor | 0.121±0.052** | 0.347±0.252 | 1.052±0.323 |
| CXCL16 | 0.165±0.055** | 0.627±0.454 | 0.991±0.811 |
| Cyr61 | 0.140±0.045** | 0.175±0.117* | 0.925±0.293 |
| DLL4 | 0.069±0.007*** | 0.082±0.037** | 0.962±0.889 |
| DPPIV | 0.604±0.068* | 0.558±0.179 | 0.768±0.220 |
| EGF | 0.148±0.035** | 0.197±0.111* | 1.258±1.149 |
| Endoglin | 0.441±0.083* | 0.532±0.207 | 1.132±0.191 |
| Endostatin | 0.739±0.020** | 0.791±0.072 | 1.019±0.094 |
| Endothelin-1 | 0.151±0.065** | 0.498±0.340 | 1.014±0.541 |
| FGF-1 | 0.500±0.086* | 0.486±0.193 | 0.943±0.144 |
| FGF-2 | 0.139±0.031** | 0.397±0.300 | 0.998±0.732 |
| FGF-7 | 0.130±0.038** | 0.104±0.059** | 0.785±0.688 |
| CX3CL1 | 0.111±0.039** | 0.108±0.063** | 0.994±0.940 |
| HB-EGF | 0.104±0.027*** | 0.178±0.119* | 0.965±0.847 |
| HGF | 0.251±0.111* | 0.274±0.050** | 1.665±1.369 |
| IGFBP-1 | 0.603±0.138 | 0.728±0.136 | 1.176±0.133 |
| IGFBP-2 | 0.141±0.049** | 0.358±0.186 | 1.049±0.691 |
| IGFBP-3 | 0.826±0.081 | 0.931±0.044 | 1.115±0.123 |
| Leptin | 0.132±0.039** | 0.168±0.089* | 0.891±0.747 |
| MMP-3 | 0.882±0.074 | 0.972±0.064 | 1.123±0.084 |
| MMP-8 | 0.227±0.014*** | 0.542±0.336 | 1.068±0.814 |
| MMP-9 | 0.808±0.084 | 0.870±0.118 | 1.249±0.059 |
| NOV | 0.270±0.070** | 0.286±0.187 | 1.294±0.178 |
| Osteopontin | 0.797±0.056 | 0.769±0.077 | 0.936±0.055 |
| PD-ECGF | 0.173±0.039** | 0.226±0.095* | 0.779±0.671 |
| PDGF-AA | 0.136±0.039** | 0.288±0.212 | 0.872±0.660 |
| PDGF-AB/BB | 0.207±0.063** | 0.151±0.076** | 0.954±0.690 |
| Pentraxin-3 | 1.468±0.321 | 1.512±0.293 | 1.356±0.518 |
| Platelet Factor 4 | 0.934±0.034 | 1.089±0.051^#^ | 1.071±0.048 |
| PlGF-2 | 0.399±0.012*** | 0.772±0.344 | 1.072±0.784 |
| Prolactin | 0.236±0.065** | 0.406±0.196 | 1.288±1.199 |
| Proliferin | 0.313±0.065** | 0.582±0.229 | 1.576±1.235 |
| Serpin E1 | 1.097±0.047 | 1.111±0.187 | 1.390±0.224 |
| Serpin F1 | 0.652±0.252 | 0.405±0.199 | 0.519±0.288 |
| Thrombospondin-2 | 0.262±0.063** | 0.092±0.015** ^#^ | 0.918±0.070 |
| TIMP-1 | 0.581±0.213 | 0.386±0.215 | 0.930±0.679 |
| TIMP-4 | 0.364±0.067* | 0.604±0.187 | 0.492±0.279 |
| VEGF | 0.536±0.188 | 0.683±0.022** | 0.246±0.090* |
| VEGF-B | 0.297±0.076* | 0.351±0.138* | 0.952±0.879 |

Values are mean±SEM; n=3 per each experimental group. *p<0.05, **p<0.01 and ***p<0.001 vs. control; ^#^p<0.05 vs. LPS.

*Tie-2 receptor antagonism by NLLMAAS*

In previous studies, the peptide NLLMAAS inhibited Ang-1 effects both *in vitro* and *in vivo* [1-2]. For the purpose of our study, we administered NLLMAAS (330µg) in three different regimens: (i) intravenous co-administration with MAT.Ang-1, (ii) intraperitoneal post-treatment to MAT.Ang-1 at 3, 4 and 5 hrs, (iii) intraperitoneal pre-treatment 1 hr before MAT.Ang-1 *plus* post-treatment at 3 hrs. As shown in Supplemental Figure 3, all NLLMAAS regimens induced macromolecular leak, starting at 22 hrs and peaking between 23 and 24 hrs of the experimental protocol.

**Supplemental Figure 3:** Macromolecular leak in controls, LPS, MAT-Ang1 and MAT-Ang1+NLLMAAS group. Data are mean±SEM.

**SUPPLEMENTARY REFERENCES**

1. Tournaire R, Simon MP, le NF, Eichmann A, England P, Pouyssegur J: **A short synthetic peptide inhibits signal transduction, migration and angiogenesis mediated by Tie2 receptor**. *EMBO Rep* 2004, **5**:262-267.
2. Wu D, Gao Y, Chen L, Qi Y, Kang Q, Wang H, Zhu L, Ye Y, Zhai M: **Anti-tumor effects of a novel chimeric peptide on S180 and H22 xenografts bearing nude mice**. *Peptides* 2010, **31**:850-864.
